# ‘The objective was about not blaming one another’: a qualitative study to explore how collaboration is experienced within quality improvement collaboratives in Ethiopia

**DOI:** 10.1186/s12961-023-00986-8

**Published:** 2023-06-13

**Authors:** Zelee Hill, Dorka Keraga, Abiyou Kiflie Alemayehu, Joanna Schellenberg, Hema Magge, Abiy Estifanos

**Affiliations:** 1 Institute for Global Health, University College London, Guilford St, WC1N 1EH London, United Kingdom; 2 Department of Reproductive, Family and Population Health, School of Public Health, Addis Ababa University, Addis Ababa, Ethiopia; 3 Institute for Healthcare Improvement, Addis Ababa, Ethiopia; 4 Department of Disease Control, London School of Hygiene and Tropical Medicine, Keppel Street, London, WC1E 7HT United Kingdom; 5 Division of Global Health Equity, Brigham and Women’s Hospital, 75 Francis Street, Boston, MA 02115 United States of America

**Keywords:** Quality improvement, Collaboration, Low-and middle-income countries, Ethiopia

## Abstract

**Background:**

Quality improvement collaboratives are a common approach to improving quality of care. They rely on collaboration across and within health facilities to enable and accelerate quality improvement. Originating in high-income settings, little is known about how collaboration transfers to low-income settings, despite the widespread use of these collaboratives.

**Method:**

We explored collaboration within quality improvement collaboratives in Ethiopia through 42 in-depth interviews with staff of two hospitals and four health centers and three with quality improvement mentors. Data were analysed thematically using a deductive and inductive approach.

**Results:**

There was collaboration at learning sessions though experience sharing, co-learning and peer pressure. Respondents were used to a blaming environment, which they contrasted to the open and non-blaming environment at the learning sessions. Respondents formed new relationships that led to across facility practical support. Within facilities, those in the quality improvement team continued to collaborate through the plan-do-study-act cycles, although this required high engagement and support from mentors. Few staff were able to attend learning sessions and within facility transfer of quality improvement knowledge was rare. This affected broader participation and led to some resentment and resistance. Improved teamwork skills and behaviors occurred at individual rather than facility or systems level, with implications for sustainability. Challenges to collaboration included unequal participation, lack of knowledge transfer, high workloads, staff turnover and a culture of dependency.

**Conclusion:**

We conclude that collaboration can occur and is valued within a traditionally hierarchical system, but may require explicit support at learning sessions and by mentors. More emphasis is needed on ensuring quality improvement knowledge transfer, buy-in and system level change. This could include a modified collaborative design to provide facility-level support for spread.

## Background

With the recognition that improvements in quality of care are needed to further reduce maternal and neonatal deaths [1], there has been increased implementation of quality improvement interventions in low and middle-income settings where groups of workers problem solve around deficient practices. Despite the growing implementation the impacts of these interventions in low and middle-income settings has been inconsistent, with small numbers of low quality evidence studies [2, 3]. Whilst there are several quality improvement approaches, we focus on Quality Improvement Collaboratives (QIC), which are a common approach to bridging the quality gap [4–6]. They work on the premise that collaboration enables improvement as it facilitates learning and action more effectively than didactic methods [7]. The evidence on the impact of QICs in low and middle-income settings is varied with some evidence of sustained impacts [8], but the most rigorous studies show little impact on patient outcomes and variable impacts on other outcomes, unless QICs and training are combined [4]. The variability in impacts suggests that transferability may be context specific, and that we need a better understanding of how core components of QICs are influenced by context [9–12].

Collaboration is a core component of QICs, and it occurs within a facility as quality improvement teams work together to address an improvement issue through ‘Plan-Do-Study-Act’ cycles. This continuous quality improvement, which was adapted from industry, underlies many quality improvement approaches [7, 13, 14]. Particular to QICs is that collaboration also occurs across facilities at ‘learning sessions’ where QI teams from several facilities meet to share results, reflect on lessons learnt and offer support and encouragement; thus accelerating the pace of improvement [7]. Teams also learn from experts who facilitate learning sessions and mentor QI teams through regular facility visits [15].

Collaboration can be advantageous in that it can cultivate a community-based approach to learning and problem solving where problems and solutions becomes collective responsibilities. It can foster innovation, generate ownership and a shared identify, exert pressure to model those performing well, improve co-operation and motivation, reduce resistance to change and encourage horizontal relationships. This collaborative advantage may be weakened in hierarchical contexts, with poor leadership, and through inertia caused by a lack of visible improvement, a lack of shared goals and when participants are driven by self-interest [16, 17].

Despite its centrality, few studies of QICs report on the collaborative element [13], and even fewer have focused on understanding how it works in practice [17]. Processes related to collaboration: lesson sharing, teamwork, competition, and peer pressure have been identified as mechanisms through which QICs works, but studies are mainly from high income settings [12]. We located six studies from low and middle income settings that met the criteria for utilizing a QIC approach [18] that mentioned collaboration and none that focused on it. Four studies found that collaboration was weak due to passive teams, a lack of shared responsibilities, hierarchical structures and top-down leadership and peer inertia due to skills remaining in the QI team rather than being spread to others [19–22]. In one study learning sessions were less valued than mentor visits suggesting that their collaborative advantage was not realized [19]. Two studies reported positively on collaboration, one multi country study concluded that the social dynamics of collaboration contributed to improvement, which would not have occurred if teams were working independently [8] and another that collaboration across facilities doubled during implementation of the QIC [23].

Many low and middle income settings are traditionally more hierarchical than high income settings, for example with more controlling management styles, formal rules and defined and rigid responsibilities [22, 24, 25] and little is known about how collaboration with QICs functions in such settings. In this paper we explore how collaboration is experienced within QICs in Ethiopia, which has a historical context of authoritarian regimes and of hierarchy and bureaucracy that influence the structure and function of the health system [26], we aim to understand how the collaborative element of QICs transfers from high to low income settings and to learn lessons for implementers about this key mechanism.

## Methods

The study was conducted in Oromia and Amhara regions of Ethiopia in *woreda* (districts) where the Institute for Health Care Improvement (IHI) was supporting the Federal Ministry of Health to implement QICs to improve the coverage and quality of ante-natal, delivery and postnatal care including the implementation of three ‘clinical bundle’, the details of which are reported elsewhere [27]. These QICs were part of a prototype phase prior to a larger scale up [27]. In the prototype *woreda* each health center and its health posts formed facility level QI teams as did each hospital. Each QI team sent representatives to four IHI facilitated learning sessions, held over a 15 month period. In these sessions participants learnt QI methods, developed QI projects, received mentoring and clinical updates, and shared their project implementation experience and challenges. In between the learning sessions facilities implemented Plan-Do-Study-Act cycles on areas requiring improvement and IHI mentors made visits to provide QI and clinical mentorship [28]. Mentors also worked with the teams to improve the data quality [29]. The inclusion of hospitals, health centers and health posts in the same collaborative aimed to facilitate the formation of a network with collective responsibility over a shared catchment area.

In each *woreda* we selected the hospital and two health centres—one in a remote area accessed by a rough road and one less remote accessed by a paved road. The health centres were selected to be in a typical area for the *woreda* in relation to economy and ethnicity. Within each health centre we interviewed 5–8 staff members including the health centre/department head, the Maternal and Child Health (MCH) focal person, the Health Information Technician, Health Extension Workers and other staff involved in QI. We purposively selected respondents who had, and had not, attended learning sessions; but all respondents were involved in QI in some way either by being on the QI team or implementing change ideas. We also interviewed the QI mentors. Interviewers were usually able to identify and approach potential respondents themselves at the facility, but when needed respondents were asked to name others involved in QI who were then approached and invited for interview in a private place in the facility. No one refused to participate, but we frequently needed to re-schedule interviews to ensure we did not interrupt clinical practice.

Data were collected by six interviewers (see Acknowledgments) who were trained for a week and had either a Bachelors or Masters in a health-related subject and experience with qualitative data collection. Most were qualitative consultants with over 5 years of experience and five were female and one was male. Data were collected between July–August 2018 through in-depth interviews in local languages. Interviewers used pre-tested semi-structured guides which included questions on how the QI team and the collaborative was being implemented, the acceptability and perceived effectiveness of the learning sessions and the mentoring, successes and challenges with the QI team and the collaborative and any changes in work life related to the QIC. This content reflected a phenomenological focus of the study to draw on participants’ lived experiences and descriptions. Interviews with health centre staff lasted between 45 and 90 min and those with mentors up to two and a half hours.

Interviews were digitally recorded and field notes taken, interviews were transcribed and translated into English by the interviewers as soon after the interviews as possible, which was usually between 1–2 days of the interview. Transcripts were reviewed by the senior researchers (ZH and AE) as soon as they were completed, who provide feedback to enhance reflexivity and data quality and held daily de-briefs to discuss emerging themes, the impact of interviewer characteristics on data collection, saturation and any changes to the interview guide.

**Fig. 1:**
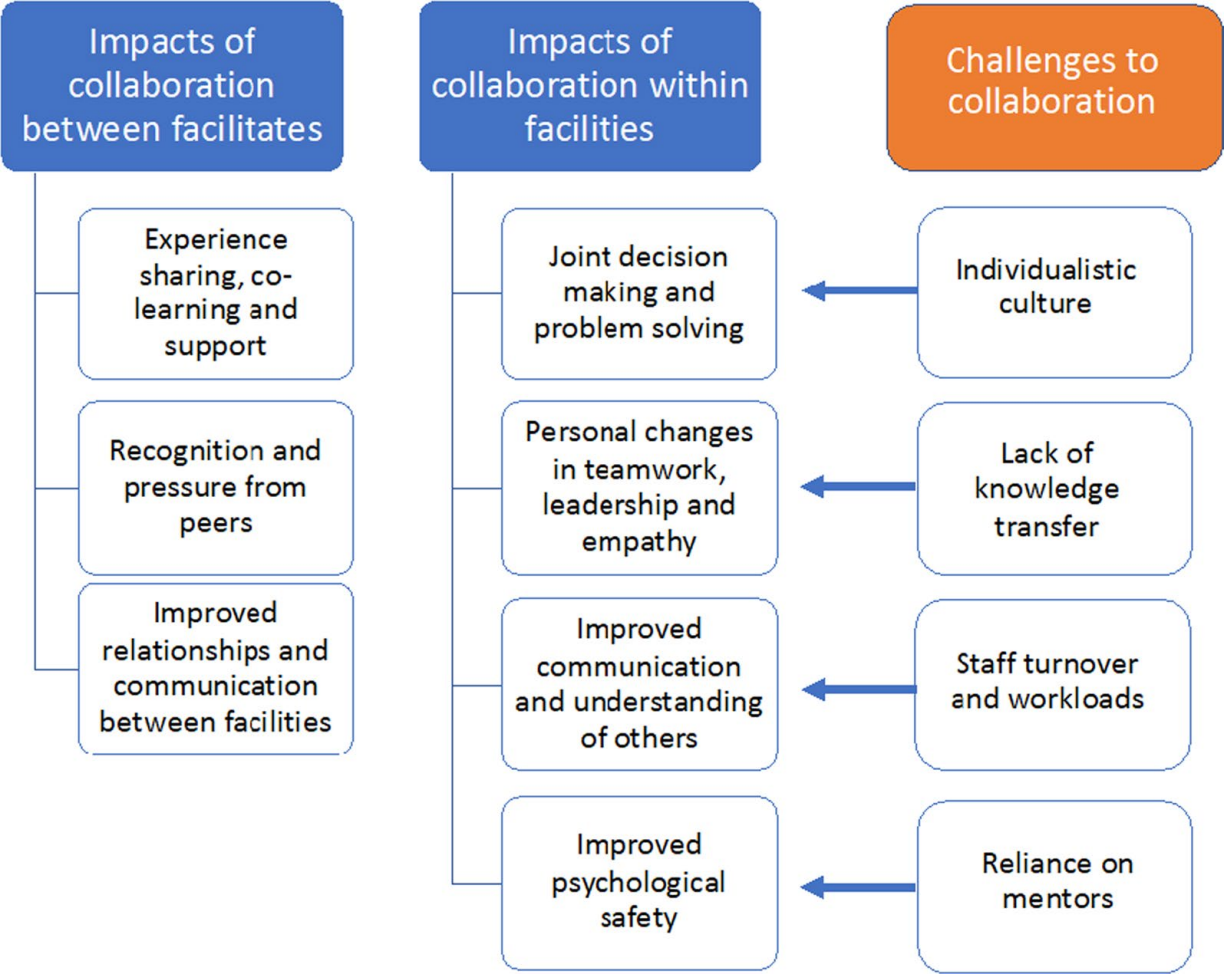
Themes related to the impact and challenges of the collaborative component of QICs

Data were collected as part of a larger project to understand implementation of QICs [30]. Interviews were hand coded thematically by the lead author (ZH) [31]. Thematic coding began through reflexive discussions with the interviewers during data collection around key findings. Transcript were then read for familiarization and to examine the data set as whole. Each transcript was then re-read and coded in relation to experiences, impacts and challenges of between and within facility collaboration, these codes were then grouped into meaningful themes, which were iteratively reviewed with new themes added and similar themes merged as appropriate. Findings were then interrogated to explore patterns, links and contradictions in the data. The final coding tree is shown in Fig. 1.

### Ethics

Ethical approval was obtained from University College London Research Ethics Committee Office for the Vice Provost Research (Project ID: 4063/002). The Ethiopian Public Health Association (EPHA) Internal Scientific and Ethical Review Committee (ISERC) provided an ethical waiver as they considered this part of the QI programme evaluation (EPHA/OG/5046/17). Participants were given information through an information sheet which included the aims of the study, how they were selected, interview procedures and length and were then asked to consent to participate in the study if they wished to take part. The methods adhere to the COREQ reporting guidelines for qualitative research.

### Study setting and respondent characteristics

The hospitals and two of the health centres had good accessibility (surfaced road) although one of these had accessibility issues for some communities in the rainy season due to a river that was difficult to cross. One of the two more remote health centres had accessibility issues throughout the year and the other in the rainy season. All facilities reported some issues with equipment, medicines or laboratory reagents, two health centers reported issues with continuous electricity and one only had access to a rainwater tank. The characteristics of the health facility respondents can be found in Table 1, the three mentors were all male.

**Table 1:** Background characteristics of the health facility study participants (*n* = 42)

Characteristics	Frequency*
Sex	
Female	26
Male	16
Facility type	
Hospital	11
Health centre	31
Job title	
Head of health centre/department	7
Maternal and child health (MCH) focal person	4
Midwife/nurse	11
Health officer	3
Health information technician (HIT)	4
Laboratory technician	1
Health extension worker (HEW)	12
Attended learning sessions	
Yes	27
No	15
Time at the facility	
< 1 year	3
1–4 years	23
5–9 years	12
≥ 10 years	2
Not known	2

## Results

Themes were divided into the impacts of the QIC on between facility collaboration (through the learning sessions) and on within facility collaboration (within the facility QI team and with those outside of the team). Themes related to the impacts of collaboration and challenges to/of collaboration were identified, and are shown in Fig. 1. The results begin with a general description of who was involved in the learning sessions and the facility QI teams and how they were selected, followed by results for each of the identified themes.

### Team composition and selection

Facility QI teams ranged from 4 to 9 people who were usually selected by the facility or departmental head. In the health centres the team was usually multi-disciplinary including the facility head, MCH focal person, midwives, out-patient department focal person, and the laboratory and/or pharmacy focal persons. Health Information Technicians were included in two of the four health centres. In the hospitals the teams were comprised of staff providing clinical care. In one hospital the QI team spanned MCH, delivery and the neonatal intensive care units, while the other hospital had an existing structure for QI with each department having a separate team.

Most facilities reported that IHI gave guidance on who should attend learning sessions, and this did not always correlate with who was on the facility QI team. For health centres this was usually the facility head, MCH head, Health Information Technician, and Health Extension Workers. In the hospitals the heads of relevant departments attended, this was usually the MCH head, neonatal intensive care unit head, head of the delivery ward, Health Information Technician and the chief executive officer and/or medical director. In some facilities midwives and Health Extension Workers were sent in rotation as this was seen as fairer, while in others the same staff attended all sessions.

### Collaboration between facilities

#### Experience sharing, co-learning and support

Learning sessions were reported as collaborative events with broad participation through group discussions and presentations. This collaboration allowed for experience sharing and co-learning in relation to what had worked and not worked, the cause of low performance and plans and ideas: *‘…. the way they were teaching was very good….all care health facilities were presenting what they were working on and they were exchange their experiences. They were presenting their challenges and they were presenting their successes, and they were teaching in that way… people were actively participating….. in all individuals there are ideas and solution for solving problems’* (Head Hosp1).

Respondents were used to a blaming and non-motivational environment in their routine *woreda* meetings, which they described as an ‘evaluation’, where they feared being punished or insulted for poor performance or for raising queries. Respondents contrasted this blaming environment to the open and accepting environment at the learning sessions which focused on understanding and solving problems rather than blaming individuals. This facilitated collaboration and idea sharing as there was no fear of repercussions for speaking out: ‘*It was criticizing each other that was happening before, but the learning session is to understand the problem first rather than criticize….. The woreda office may say ‘Why you did not do that?’….. They* [in learning sessions] *say what is holding you back?…. Everyone can raise an idea* [at the learning sessions]*… in the woreda meeting if you do not accept their* [*woreda* officials’] *idea they may say get out! Here it is not like that… everyone can raise his problem or idea freely*’ (Head F1).

#### Recognition and pressure from peers

The collaborative nature of the learning sessions meant that facilities saw what others were doing and commented on each other’s performance. Facilities that were doing well were recognised for their good performance and suggestions were given to those performing less well. The public recognition of good performance made facilities proud of their achievements: *‘All people appreciate us, they say X health center does the best work… Our presentation was perfect, we had high performance… all people appreciated it’* (MCH focal F1). Although a key theme was the supportive and non-blaming environment at the learning sessions a few respondents felt feelings of shame when they performed less well than others which pressured them to perform better: ‘*We felt shame when we returned to facility… …. We have learnt a lot of things….. it made us regret and learn from our problems… We failed to achieve the plan before. We discussed what has to be done to achieve the plan…. We are trying to do our best in order to provide quality service’* (HEW F1).

#### Improved relationships and communication between facilities

Meeting together in an open environment increased the links between facilities, this included increased connections and communication between different levels of provision (i.e. between hospitals, health centres and health posts) which were reported as being weak before the QIC. For example, solving problems such as ante-natal care attendance required discussions between the health centre and their health posts which allowed for a greater understanding of each other’s work: *‘The relationship between the PSU* [health center] *and the health post was very light… when the project* [QIC] *needed to work on the ANC or delivery…. They were working from bottom level *[health posts] *so this learning session connected these people…. The relationship among one health center with other health center it* [learning session] *makes them strong’* (Mentor 2). This increased connection also led to the provision of practical support such as the exchange of drugs and materials and improved referral links: *‘If our health centre doesn’t have syphilis test reagents, X or Y health centre gives them and when it comes we return it to them … So, this program makes the relationship between different health facilities strong’* (HIT F3).

Despite reports of the benefits of improved links a few hospital respondents felt that combining hospitals and health centres in learning session was detrimental to learning as there was little a hospital could learn from a health centre and would have preferred separate sessions: *‘We can’t learn from them* [health centers]*… it would be good if hospital is compared with hospital because we have to learn from one another’ *(QI focal Hosp2).

### Collaboration within the facility

#### Joint decision making and problem solving

Mentors reported explicitly teaching teamwork skills in the learning sessions , as they recognized that teamwork was important for the QI teams to function: *‘If you are working as a team you plan as a team, you identify ideas as a team, test ideas as a team and you analyse as a team…. We are promoting that in the learning sessions. We let them do that when they return back in their facilities’* (Mentor 1). Respondents reported that teamwork and collaboration occured within the QI team, at least at the beginning when teams were stable, with decisions made through discussion and active participation and team members considering and learning from each other’s ideas. The joint decision-making enhanced ownership, shared responsibility and feelings of fairness and transparency: *‘In meetings we discuss equally. For example, a man can bring an idea. But, then we all talk about the idea, if it is correct and it will solve the problems, then everybody will be convinced. It is not just the opinion of head of the health centre…. In our health centre, only the best idea is selected. And our participation is also equal’* (Midwife F1).

This team feeling was enhanced by seeing the impacts of the change ideas, with team members getting satisfaction from contributing to this change, which enhanced their work ethic: *‘Before, I don’t want to collect and analyse the data... But, now I am happy…. I have contributed to something'* (HIT F1). Conversely, teamwork and commitment was weakened when there was little change, especially when this was due to issues outside of the teams control such as a lack of resources or workforce: *‘…at first, the quality team members were happy. But now their emotions are not good as before. There is a problem attending our meetings…. the work we are doing is getting smaller. At first, there was a good sense of moral and a good activities… to tell you the truth…there is no activities like before…. now, they don’t want it ….they want spend their time for their own personal interest. Some members argue that there is no change and there is no progress in any project… so why we suffer?’* (Midwife F1).

#### Personal changes in teamwork, leadership and empathy

Those who had attended learning sessions reported changes in their teamwork skills and felt more empowered and more able to speak up, listen, be empathetic and have the ‘confidence’ to admit when they don’t know something: *‘I should be learning from my mistakes instead of criticizing* [others].*….. Insulting doesn't change man….If you talk to people with love, they will do good things…. I have learnt politeness…’* (MCH focal F2). Some facility/department heads reported changing their leadership style to be less blaming as a result of being in the QIC: *‘Before when a person did something wrong he was disciplined or fired, we were not trying to know the reason…. Now I talk positively; leadership is showing by doing not pushing others to do what they do not want to do’ *(Head F1), and adopted a more collaborative work style: *‘He* [head] *started their routine evaluation as a learning session style … It is a big success to find a health centre head who starts working the same way as in a learning session’* (Mentor 1). These personal changes spilled over into respondents’ personal lives: *‘…. It helps to see other options, it shows there are many alternative ways when something happens in life. If I have a problem it helps me not to think there is only one solution… it helps not only for work but also for my personal life'* (Head F1).

#### Improved communication and understanding of others

QI teams reported improved communication with others in the facility, particularly in relation to problems such as stockouts. This was attributed to having a greater commitment towards quality and taking an active rather than passive approach to their work: *‘It [the QIC] makes me have commitment….. when there is shortage of something, it makes us communicate with laboratory or pharmacy at an early stage. Previously … if there is iron, we give it …. but if there is not iron we don’t feel anything* [are not concerned by it]’ (MCH focal F3).

#### Improved psychological safety

Some respondents also reported an increased ability to correct mistakes and share problems or concerns without fear due to greater connections, improved relationships and a feelings of shared responsibility: *‘Now we comment on each other and correct each other… we do not hide anything…. Since we have a close relationship with the midwives we can tell them what the communities complain of and help them to correct it’* (HEW F4).

### Challenges to collaboration

A change in collaboration was reported in all facilities, but the extent varied by facility and over time. Key challenges were:

#### Individualistic culture

Collaborative teamwork was new for many respondents, and these new skills were gained within a system that focused on individual responsibilities and achievements. This was a challenge to collaboration in that there could be a lack of team spirit and defensiveness rather than an open approach to discussions: *‘People are not familiar with team work…. Individual work is promoted…. To be honest it was very challenging to bring that team spirit…When you ask them to work as a team it was not very effective. People start defending themselves… they are familiar with evaluation not team work….. one person sits and writes something and tells you that they work as a team’ *(Mentor 1). One of the mentors lamented that this focus on individual work and responsibilities contributed to a lack of system level changes, with QICs mainly working at an individual level and weaknesses in the broader systems constraining longer term impacts: *‘The system we have is fragile…. It is only the people who are working not the system. The work is done by the people not the system… You have seen impacts *[of QI] *in different places, you have seen a kind of sparkle but immediately it vanished. This is because it is not the system which is working it is the people’* (Mentor 1).

#### Lack of knowledge transfer

When those who had attended learning sessions returned to the facility QI knowledge was rarely transferred to those who had not attended. Knowledge transfer was hindered by a lack of time, the perceived complexity of QI in relation to its specific terminology and methodologies and because of a lack of interest among those who had not attended. In some facilities knowledge transfer was not attempted at all, but in others it was tried but was found to be difficult: *'We selected one person from the QI team, and that person gave a kind of orientation *[to others in the facility] *about quality …. what is quality means? What change idea means? What we plan to do…when we tried to explain … they don’t understand about it. We tried to orient them, but they had a “what was that all about” kind of look’* (MCH focal F1).

Not all members of facility QI teams attended learning sessions which, given the lack of knowledge transfer, meant that team members often had varied levels of QI knowledge and consequently varied participation in discussions and decision making, and varied ownership of the change ideas and motivation to implement and monitor them. In some teams those who had not attended the learning sessions lacked, or were perceived to lack, the skills for meaningful contribution: *‘QI is scientific and it takes time until you fully understand it, it is very challenging…. it *[decision making] *is dominated by me.… they* [others in the team] *were not trained….I cannot say the level of participation is good. The participation is not good’ *(QI focal Hosp2).

There was also a lack of spread outside of the QI team and in the hospitals, where learning session attendants were spread across departments, some non-attendants knew nothing about the QIC except that they had been told to change their practices: *‘They* [who attended learning sessions] *never teach us what it is about when they return to the facility. They never share information, they do not tell us anything’* (Midwife F1). There was a perception among those who had not attended learning sessions that attendants received financial benefits; this led to some resentment towards those who attended and a resistance to implementing change ideas that required them to take on more work or modify their roles. This, combined with an individualized focus on roles and responsibilities, led to a feeling that QI related work should be the remit of the QI team: *‘There are workers who say: That person who was trained should work… I was not trained… I have no understanding about this and I do not work it’ *(Head 3). Many respondents felt that QI would work better and be more sustainable if there was wider participation with more people trained or directly included in the QIC and a more inclusive approach:* ‘The team is good but it does not involve all, this is the main limitation, there is only one person from MCH and one from delivery….but there are around nine workers in this delivery department…. I don’t think only one person can bring improvement… it is better it they work by involving all the entire workers… it is not participatory’* (Midwife Hosp1). This feeling was echoed among those outside of the MCH department, e.g. QI team members from the laboratory, pharmacy, out patients, pediatrics and health information, as they usually did not receive mentor visits and thus sometimes felt overlooked and unsupported.

#### Staff turnover and workloads

Structural issues directly affected the teams’ ability to meet and function and the sustainability of the QIC. Facility QI team meetings were often suspended or reduced during times of high workloads, or gradually reduced over time due to competing priorities: *‘At the beginning, we all had active participation. When there is workload, what we were working on* [QI] *gets reduced’* (NICU head Hosp2). Staff turnover was also a challenge as team relationships and dynamics were affected by membership changes, experience and knowledge were lost and it took time and effort to re-establishing the team. This was a particular problem in the facility where the facility head, who strongly supported the QIC, resigned: *‘Their* [X facility] *project used to run very well…. There was no staff which I like more than X staff, I enjoyed working with them, they frequently practiced the PDSA model, and they frequently prepared run charts, they have done everything very well…. but the head resigned, and we again established QI again. When we do that the staffs became new, so we can’t move forward as we expected’ *(Mentor 1).

Collaboration was further undermined by a culture of dependency where facilities looked to NGOs to bring material support and be an external driver, rather than to provide support for their own problem solving: *‘They *[facilities] *ask you* [mentors] *for infrastructure not the support you give. That is the habit when it is an NGO, they look for big material support… the attitude of dependency was very high…. their ideas are associated with materials. Their mind was occupied by these things’ *(Mentor 2). Previous experiences with NGO projects led to a perception that the QIC was a short-term project, with some participants reporting that it had terminated when it transitioned from IHI to *woreda *support. The perception that the QIC was an externally led project with an end led to reduced ownership:* 'At times, it was the IHI project. In a sense…. people thought we were doing this for IHI’* (Head F2).

#### Reliance on mentors

In most facilities there was a reliance on the mentors to drive the QI processes: '*I don’t think that the team can continue ….unless somebody comes and initiates us, the meetings will be discontinued’* (HEW F3). Mentors visited frequently, weekly in some cases, and were described as having unique skills and characters and were extremely well regarded: *‘The*y [mentors] *are very good….. I never saw such a strong person. … He bothers about our mothers more than us’ *(Head F3). Mentors modeled a collaborative approach and encouraged deep thinking about problems rather than focusing on immediate solutions which was a common problem: ‘*They* [QI team] *need support from us* [mentors] *especially on the problem analysis…. they try to find an immediate solution to the problem…….. There answers were “we don’t have money”…. “the community doesn’t have awareness”…. When they go in detail, they find the source of the that problems…. There was big support on that’ *(Mentor 2).

Mentors also supported collaboration by ensuring the facility QI team met together, either by convening meetings themselves or checking and motivating the team to keep active through advice, encouragement and praise. Their hands-on approach was sometimes modeled by those in leadership roles at the facilities: *‘Most of the time the director joined me* [mentor] *and checked the delivery room. Previously, he was not observing the problems of the midwife’s and what the delivery room looked like…. when I looked at it, their *[director and midwives] *relationship was becoming strong’* (Mentor 2).

## Discussion

We found that learning sessions were collaborative, with facilities learning from each other and forming new relationships in an open and non-blaming environment. Equipping participants with teamwork skills was an aim of the sessions, and participants noted that the atmosphere and approach in the learning sessions was new and different. The sessions also provided pressure to perform well with poor performers feeling shame, and high performers motivated by appreciation and recognition. Within facilities those who attended the learning sessions continued to collaborate with each other, although this was often perceived as driven by mentor visits. However, QIC was sometimes alienating for those who did not attend sessions due to its perceived complexity and a lack of transfer of knowledge and skills. This led to unequal participation and more hierarchical decision-making in some teams. Those not in the facility QI team who were asked to change their practices sometimes felt resentment and resistance, with a perception that change ideas should be the remit of the QI team and that the QIC should be more inclusive. Respondents who attended learning sessions reported individual changes which would improve their ability to collaborate such as being empowered to speak up, listen, and be empathetic; but overall there was a lack of system level change. Challenges to collaboration included unequal participation, poor staff capacity, small numbers attending learning sessions, high work loads, staff turnover, a culture of dependency and a view of NGO projects being short term.

Collaboration was an important element of the QIC, and was possible in a hierarchical setting that traditionally blames individuals [30, 32], within the right environment respondents no longer felt worried about being insulted, blamed or criticised for poor performance or for raising ideas or problems. However, without a wider change in organizational culture this openness and collaboration may not be sustainable. We did not find that self-interest disrupted collaboration in the learning sessions or within the QI team or that that professional hierarchies reduced collaboration as has been found by others [17, 20], this may be because the QIC had frequent mentor visits that enabled more horizontal collaboration. This is an important finding given that a study from Nigeria found that in an effort to improve compliance and effectiveness and to be more aligned with existing organizational cultures QICs explicitly adopted a top down approach with hierarchical decision making [22]. The move to a top down approach undermines the basic principles of QICs and our study suggests that it may not be necessary if QICs explicitly foster team work and collaboration.

Engaging and collaborating with those outside of the QI team has been linked with QIC success [33], but this rarely occurred and consequently for some the QIC was alienating. This limited the potential for a sustainable culture of improvement to develop. Other studies have identified peer-inertia as an issue when a small proportion of staff are trained in QI and where knowledge transfer is limited [34]. A lack of engagement with non-QI team members is common, a systematic review of the components of QICs found only 6 of 24 studies reported engagement with non-QI team members [13], and others have also found little spread [20]. In some facilities no attempt was made to transfer knowledge, this may reflect that staff were too stretched to engage in unstructured learning, felt it was a waste of time given high staff turnover or because there were not the monetary incentives for staff to attend knowledge sharing sessions within the facility. In other facilities transfer was tried but was difficult due to the complexity of QI, suggesting that skill building among those trained was not yet strong enough for knowledge transfer. More emphasis is needed on ensuring staff outside of the QI team are engaged in QI so that QICs reduce rather than increase resistance to change. This could include a modified collaborative design which provides facility-based supports for QI spread or uses online resources or tools, rather than relying on QI teams to do it organically.

High levels of staff turnover were problematic for collaboration, this may be a particular issue where those trained in QI or who have the highest QI capacities are the most likely to be promoted elsewhere [35, 36]. For QI to be sustainable some level of stability in the team is needed [37, 38], unless QIC becomes institutionalized and known by all staff.

In some settings QI mentoring has been perceived as more valuable than the learning sessions [19], but we found that the learning sessions were highly regarded as opportunities for learning in a non-blaming environment. Mentors can play an important role in fostering a culture of improvement and collaboration [39] and we found that they imparted skills, including teamwork skills, and helped ensure QI structures and processes were followed. In some facilities there was a sense of dependence on the mentors, attributed to an overall culture of dependency and previous experiences with short term NGO supported projects. The NGO mentors were highly skilled and able to model and teach the skills needed for teamwork and collaboration to those used to a more hierarchical approach. At scale, the high quality and frequent support provided by the mentors may not be feasible [37], and the types of collaboration we found may be more limited. The prototype phase had a high level of IHI support, it was followed by a larger test of scale with a capacity transfer process to government staff, the results of the scale up are forthcoming and will provide much needed evidence of QIC implementation at scale.

Although we focus on understanding collaboration within QICs in a low-income setting, our findings also add to the limited knowledge of QIC mechanisms. Evaluations of QICs have shown varied impacts, this may be due to the influence of context on implementation and on whether mechanisms of action are triggered [9, 11]. Previous studies have identified several potential mechanisms of actions, those related to collaboration include: generating a learning and collaborative culture, improved teamwork and problem solving, and creating healthy competition and peer pressure [9, 12]. Whilst these were identified in this study, they were mostly through learning sessions with limited evidence of a learning and collaborative culture developing within facilities and a high dependence on well trained and skilled mentors for sustainability. Given the focus on collaboration we did not explore other important mechanisms such as improved capacity or improved data quality and use, nor barriers to impact identified in other low income settings such as staff shortages, high workloads and lack of supplies and equipment [20, 22, 34, 35, 40]. Whilst we show that collaboration remained a key element of the QIC, there are still unresolved questions as to whether QICs should be established in setting where facilities lack essential inputs [34, 35].

Although we purposively selected typical facilities our findings may not be transferable to other settings as context can modify how QICs are experienced and how they function [9, 12]. In addition, social desirability bias may have influenced respondents’ answers. We did not observe any major differences in themes by facility type, except for the hospital that already had a QI structure in place, but having only two hospitals in the sample limits our ability to explore differences between hospitals and health centres. Although we interviewed IHI mentors the study would have benefited from including senior IHI and Ministry of Health staff to gain their perspectives, as well as repeat interviews at a later date to explore sustainability.

## Conclusion

Collaboration was an important element of the QIC, but required support through fostering team work skills at learning sessions and through mentoring visits. It was valued by respondents, especially given previous negative experiences of a blaming culture. The QIC was sometimes alienating to those who did not attend learning sessions, which led to resentment and resistance. Challenges to collaboration included unequal participation, poor staff capacity, small numbers attending learning sessions, high work-loads, staff turnover, a culture of dependency and a view of NGO projects being for the short term. More emphasis is needed on ensuring staff outside of the QI team are engaged in QI in an inclusive way, that a core of trained staff are retained on the QI team, and that mentoring tackles any historical dependency on NGOs to foster feelings of ownership.

## Data Availability

Data are available upon reasonable request. The data are confidential considering that participants could be identified if their interviews are read in full. A formal request needs to be made and a data sharing agreement will have to be made before sharing the data.
